# A Randomized Controlled Trial of Three- versus Five-Day Artemether-Lumefantrine Regimens for Treatment of Uncomplicated Plasmodium falciparum Malaria in Pregnancy in Africa

**DOI:** 10.1128/AAC.01140-19

**Published:** 2020-02-21

**Authors:** Marie A. Onyamboko, Richard M. Hoglund, Sue J. Lee, Charlie Kabedi, Daddy Kayembe, Benjamin B. Badjanga, Gareth D. H. Turner, Nikky V. Jackson, Joel Tarning, Rose McGready, Francois Nosten, Nicholas J. White, Nicholas P. J. Day, Caterina Fanello

**Affiliations:** a Kinshasa School of Public Health, University of Kinshasa, Kinshasa, Democratic Republic of the Congo; b Mahidol-Oxford Tropical Medicine Research Unit, Faculty of Tropical Medicine, Mahidol University, Bangkok, Thailand; c Centre for Tropical Medicine and Global Health, Nuffield Department of Medicine, University of Oxford, Oxford, United Kingdom; d Shoklo Malaria Research Unit, Mahidol-Oxford Research Unit, Faculty of Tropical Medicine, Mahidol University, Mae Sot, Thailand

**Keywords:** antimalarial agents, pregnancy

## Abstract

Artemether-lumefantrine antimalarial efficacy in pregnancy could be compromised by reduced drug exposure. Population-based simulations suggested that therapeutic efficacy would be improved if the treatment duration was increased. We assessed the efficacy, tolerability, and pharmacokinetics of an extended 5-day regimen of artemether-lumefantrine compared to the standard 3-day treatment in 48 pregnant women and 48 nonpregnant women with uncomplicated falciparum malaria in an open-label, randomized clinical trial. Babies were assessed at birth and 1, 3, 6, and 12 months. Nonlinear mixed-effects modeling was used to characterize the plasma concentration-time profiles of artemether and lumefantrine and their metabolites. Both regimens were highly efficacious (100% PCR-corrected cure rates) and well tolerated. Babies followed up to 1 year had normal development. Parasite clearance half-lives were longer in pregnant women (median [range], 3.30 h [1.39 to 7.83 h]) than in nonpregnant women (2.43 h [1.05 to 6.00 h]) (*P*=0.005). Pregnant women had lower exposures to artemether and dihydroartemisinin than nonpregnant women, resulting in 1.2% decreased exposure for each additional week of gestational age. By term, these exposures were reduced by 48% compared to nonpregnant patients. The overall exposure to lumefantrine was improved with the extended regimen, with no significant differences in exposures to lumefantrine or desbutyl-lumefantrine between pregnant and nonpregnant women. The extended artemether-lumefantrine regimen was well tolerated and safe and increased the overall antimalarial drug exposure and so could be a promising treatment option in pregnancy in areas with lower rates of malaria transmission and/or emerging drug resistance. (This study has been registered at ClinicalTrials.gov under identifier NCT01916954.)

## Abstract (precis)

Artemether-lumefantrine antimalarial efficacy in pregnancy could be compromised by reduced drug exposure. Population-based simulations suggested that therapeutic efficacy would be improved if the treatment duration was increased.

## INTRODUCTION

In sub-Saharan Africa, malaria is a major cause of maternal and newborn morbidity and mortality. Efficacious antimalarial preventive and treatment regimens can reduce this significantly, but therapeutic choices are limited by concerns over possible toxicity to the developing fetus. Because of these concerns, pregnant women are commonly excluded from clinical trials (1). This, combined with the lack of adverse-event reporting systems, results in a scarcity of data on drug safety and efficacy in pregnancy. The extensive changes in maternal physiology in pregnancy alter the pharmacokinetic properties of many drugs, resulting in reduced exposures and compromised treatment efficacy (2–4). Artemether-lumefantrine is a highly efficacious artemisinin-based combination therapy approved by WHO for use in the 2nd and 3rd trimesters of pregnancy. Previous studies have shown that the standard 3-day treatment in later pregnancy is associated with lower plasma concentrations of artemether, dihydroartemisinin (DHA), and lumefantrine and more rapid elimination of lumefantrine (5–7). Low lumefantrine plasma concentrations are associated with therapeutic failure in the treatment of falciparum malaria (8–10). While the standard adult dose of artemether-lumefantrine has so far proven efficacious in high-transmission settings where pregnant women have higher levels of immunity (11), efficacy is reduced significantly in low-transmission settings where women have lower levels of immunity (9). Inadequate antimalarial prevention or treatment dosing in pregnancy risks breakthrough infection or treatment failure, respectively (12), and the consequent exposure of malaria parasites to subtherapeutic drug concentrations, which may select for drug resistance (13).

Artemether is rapidly absorbed and rapidly cleared from plasma (terminal elimination half-life of 1 to 3 h). Its major active metabolite, DHA, is formed rapidly and has a clearance pattern similar to that of the parent drug (8, 14). Artemether is metabolized into DHA mainly by the cytochrome P450 (CYP) enzymes CYP2B6, CYP3A4/5, CYP2C9, and CYP2C19 (15). Lumefantrine is absorbed slowly, followed by slow clearance from plasma (approximately 3 to 4 days up to 10 days) (14, 16). Lumefantrine, which provides most of the antimalarial efficacy, is metabolized by CYP3A4 to desbutyl-lumefantrine, which also has antimalarial activity (17). Food intake significantly increases the bioavailability of both artemether (>2-fold) and lumefantrine (approximately 16-fold) (14, 18). The absorption of lumefantrine is dose limited, so a longer treatment course has been suggested in order to improve drug exposures in pregnancy (5, 10, 18). The aim of this trial was to assess the tolerability, safety, and pharmacokinetics of an extended 5-day regimen of artemether-lumefantrine (10 doses over 5 days) compared to the standard 3-day regimen (6 doses over 3 days) in pregnant and nonpregnant African Congolese women with uncomplicated falciparum malaria.

## RESULTS

**FIG 1:**
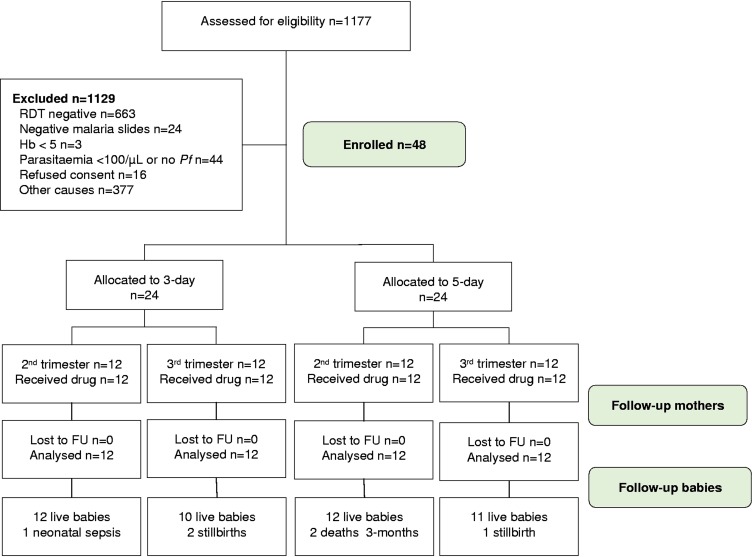
Flow diagram for pregnant women. Hb, hemoglobin; *Pf*, P. falciparum; FU, follow-up; RDT, rapid diagnostic test.

**FIG 2:**
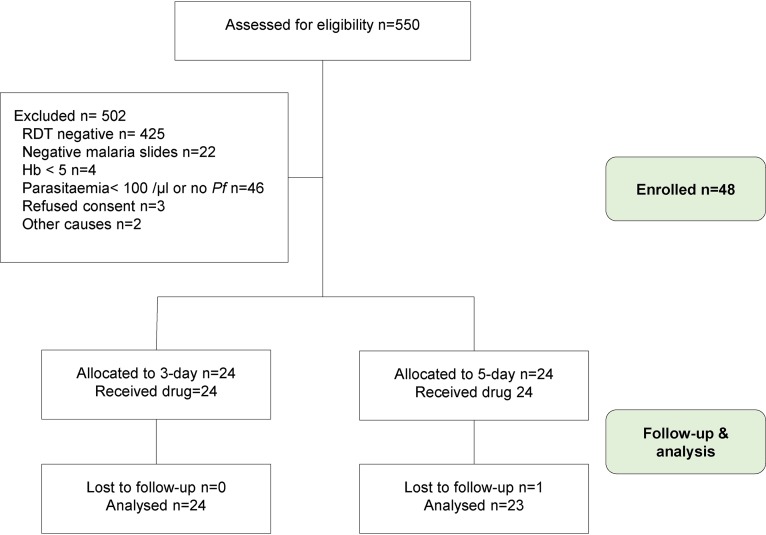
Flow diagram for nonpregnant women.

Ninety-six patients were enrolled between June 2013 and April 2014 (Fig. 1 and 2). Baseline demographic, clinical, and laboratory characteristics were similar between the patient groups (Tables 1 to 3). At presentation, the geometric mean parasitemias were similar in pregnant women (2,688 parasites/μl [range, 144 to 104,499 parasites/μl]) and nonpregnant women (2,648 parasites/μl [128 to 135,648 parasites/μl]) (*P *= 0.971), whereas the median hematocrit was significantly lower in pregnant women (29.5% [range, 24 to 36%]) than in nonpregnant women (36% [25 to 43%]) (*P *< 0.001). Pregnant women, as expected, had higher median body weights (61.5 kg [interquartile range {IQR}, 54.5, 68.8 kg] versus 53.0 kg [48.0, 61.0 kg]; *P* = 0.001) and higher median body mass index (BMI) values (24.8 kg/m^2^ [IQR, 20.8, 26.8 kg/m^2^] versus 20.5 kg/m^2^ [19.0, 23.6 kg/m^2^]; *P* = 0.0007). The median doses of artemether and lumefantrine administered by treatment arm are presented in Table S1 in the supplemental material.

**TABLE 1:** Baseline characteristics by treatment arm

Characteristic^*a*^	Value for group			
	Pregnant women		Nonpregnant women	
	3 days	5 days	3 days	5 days
No. of women analyzed	24	24	24	24
Mean age (yr) (SD)	28.5 (6.51)	26.63 (6.18)	25.7 (7.8)	28.1 (8.52)
Mean body wt (kg) (SD)	61.7 (11.6)	63.5 (9.84)	52 (6.6)	54.5 (13.7)
Median BMI (kg/m^2^) (range)	25.1 (19–30.5)	24.5 (17.3–40)	19.6 (16.9–25.3)	21.5 (17.6–35.8)
No. of women with sickle cell trait (%)	4 (16.7)	6 (25)	5 (20.8)	5 (20.8)
No. of women of parity (%)				
1	6 (25.0)	10 (41.7)		
2–4	12 (50.0)	6 (25.0)		
≥5	6 (25.0)	8 (33.3)		
Mean EGA (wk), 2nd trimester (SD)	19.2 (3.54)	18.7 (3.11)		
Mean EGA (wk), 3rd trimester (SD)	28.9 (4.42)	24.9 (5.84)		
Median temp (°C) (range)	36.4 (36.0–37.7)	36.2 (36.0–37.2)	36.5 (36–39.2)	36.4 (36–38.9)
Median hematocrit (%) (range)	29.5 (24–36)	29.5 (25–36)	37 (25–43)	35 (30–41)
Geometric mean parasitemia (no. of parasites/μl) (SD)	8,584 (14,741)	16,942 (31,587)	18,502 (36,771)	11,035 (16,846)

a EGA, estimated gestational age.

**TABLE 2:** Hematology by treatment arm

Group, parameter, and time point^*a*^	3 days		5 days		*P* value
	No. of women	Mean value (range)	No. of women	Mean value (range)	
Pregnant women					
No. of WBCs/μl					
Baseline	17	5,000 (3,600–7,200)	21	6,100 (3,400–9,800)	0.07
Discharge	17	6,300 (5,400–8,600)	21	7,600 (6,100–11,700)	<0.01
No. of neutrophils/μl					
Baseline	9	3,965 (1,325–4,622)	20	3,978 (983–6,570)	0.92
Discharge	9	4,158 (3,091–5,504)	20	4,716 (3,731–7,441)	0.13
No. of lymphocytes/μl					
Baseline	19	1,368 (275.2–2,275)	22	1,488 (638–2,660)	0.48
Discharge	19	1,867 (1,166–2,996)	22	2,054 (1,258–3,305)	0.46
Nonpregnant women					
No. of WBCs/μl					
Baseline	20	4,900 (3,200–7,400)	20	4,700 (3,200–7,600)	0.53
Discharge	20	4,600 (2,700–8,000)	20	4,900 (3,200–6,500)	0.52
No. of neutrophils/μl					
Baseline	19	2,474 (1,472–4,402)	19	2,069 (1,531–6,118)	0.35
Discharge	19	1,944 (1,116–5,372)	19	2,169 (1,440–2,836)	0.39
No. of lymphocytes/μl					
Baseline	20	1,894 (563–3,382)	20	1,667 (714–3,538)	0.59
Discharge	20	2,087 (1,037–6,160)	20	2,279 (1,263–4,248)	0.24

a WBCs, white blood cells.

**TABLE 3:** Biochemistry by treatment arm

Group, parameter, and time point	3 days		5 days		*P* value
	No. of women	Mean value (range)	No. of women	Mean value (range)	
Pregnant women					
Creatinine level (mg/dl)					
Baseline	14	0.9 (0.5–2)	20	0.8 (0.3–1.8)	0.53
Discharge	14	0.8 (0.5–1.4)	20	0.8 (0.4–1.5)	0.74
ALT level (U/liter)					
Baseline	22	16.6 (6.1–109)	22	18.8 (1.7–41)	0.61
Discharge	22	13.6 (4–40.1)	22	21.1 (5.2–57.6)	0.06
AST level (U/liter)					
Baseline	23	27 (11–163)	21	19.8 (5.8–41)	0.18
Discharge	23	24.6 (9–71.1)	21	16 (3.4–56.4)	0.04
Albumin level (g/dl)					
Baseline	23	3.7 (3–4.8)	24	3.7 (2.6–4.9)	0.57
Discharge	23	3.6 (3–4.8)	24	3.7 (3–5.5)	0.68

Nonpregnant women					
Creatinine level (mg/dl)					
Baseline	7	0.9 (0.6–1.8)	11	0.9 (0.5–1.6)	0.5
Discharge	7	0.9 (0.5–2.3)	11	1 (0.4–2)	0.65
ALT level (U/liter)					
Baseline	24	18.1 (4.6–74)	24	18 (4–97)	0.56
Discharge	24	18.8 (7.5–36.7)	24	18.2 (5.2–75)	0.43
AST level (U/liter)					
Baseline	23	27 (10.5–55.2)	23	27.1 (3.4–49.4)	0.34
Discharge	23	21 (12.3–91.6)	23	22.1 (5–95)	0.69
Albumin level (g/dl)					
Baseline	13	4.1 (3–5)	13	4.2 (3.6–6.1)	0.26
Discharge	13	4.1 (3.5–5.8)	13	4.1 (3–4.7)	0.63

### Drug efficacy.

**FIG 3:**
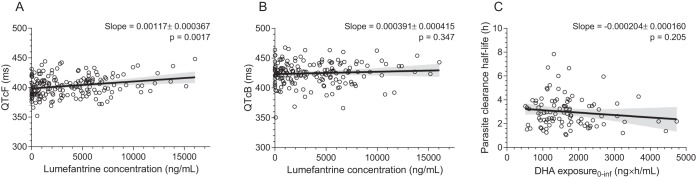
Plots of correlations between Fridericia-corrected QT intervals and predicted lumefantrine concentrations at the time of ECG measurements (A), Bazett-corrected QT intervals and predicted lumefantrine concentrations at the time of ECG measurements (B), and the parasite clearance half-life and the predicted DHA exposure (area under the concentration-time curve from 0 h to infinity) (C).The solid line is the mean regression of observed data, and the shaded areas show the 95% confidence intervals of this estimate. The slope is given as the estimated value ± standard error.

All patients cleared their initial parasitemia by day 3 in the 3-day regimen and by day 4 in the 5-day regimen. The median (range) parasite clearance half-lives (PC_1/2_) were significantly longer in pregnant women than in nonpregnant women: 3.30 h (1.39 to 7.83 h) versus 2.43 h (1.05 to 6.00 h) (*P* = 0.005). There was no significant relationship between predicted exposure (area under the concentration-time curve [AUC] from time zero to infinity) to artemether or DHA or the sum of the molar exposure to both drugs and parasite clearance half-life (Fig. 3C [data are shown only for the relationship between DHA exposure and parasite clearance half-life]). The same was seen if investigating the correlation between early exposure (sum of the molar exposure to artemether and DHA from 0 to 4 h and from 0 to 6 h) and parasite clearance half-life (data not shown).

The initial prevalences of gametocytemia were 8.3% (4/48) in pregnant patients and 14.6% (7/48) in nonpregnant women; in 6 women, gametocytes were observed on day 1 but not at inclusion (3 in each arm). There were no differences in gametocyte positivity rates between the two arms (data not shown). Gametocytes persisted in the peripheral blood for up to 2 weeks posttreatment.

In the pregnant group, the uncorrected cure rates by day 42 were 90.9% (95% confidence interval [CI], 70.8 to 98.9%) following 3 days of treatment and 90.0% (95% CI, 68.3 to 98.8%) following 5 days of treatment (*P* = 0.92). In the nonpregnant group, the uncorrected cure rates by day 42 were 87.5% (95% CI, 67.6 to 97.3%) following 3 days of treatment and 95.7% (95% CI, 78.1 to 99.9%) following 5 days of treatment (*P* = 0.32) (Table 4). The PCR-corrected clinical and parasitological response was 100% in both treatment arms, including one patient with a day 7 lumefantrine level of <280 ng/ml.

**TABLE 4:** Efficacy by treatment regimen at day 42

Outcome	No. (%) of women			
	3 days		5 days	
	Pregnant	Nonpregnant	Pregnant	Nonpregnant
Allocated	24	24	24	24
Deliveries before day 42	2		4	
Lost to follow-up	0	0	0	1 (4.2)
Evaluable	22 (91.7)	24	20 (83.3)	23 (95.8)
Early treatment failure	0	0	0	0
Late clinical failure	0	0	1 (5.0)	0
Late parasitological failure	2 (9.1)	3 (12.5)	1 (5.0)	1 (4.3)
Adequate clinical and parasitological response (ACPR)	20 (90.9)	21 (87.5)	18 (90.0)	22 (95.7)
New falciparum infection	0	3	2	1
Recrudescence (P. falciparum)	0	0	0	0
Undetermined PCR result	2	0	0	0
ACPR PCR corrected	20 (100)	24 (100)	20 (100)	23 (100)

### Fever clearance time.

At admission, 12.5% (6/48) of nonpregnant patients had fever, compared to 2.1% (1/48) of pregnant patients (*P* = 0.111). In addition, 62.5% (30/48) of nonpregnant patients had a history of fever prior to admission, compared to 46% (22/48) of patients in the pregnant group (*P* = 0.101). Fever resolution was rapid and similar in the two groups. Within 24 h, all women were afebrile.

### Tolerability and adverse effects.

By day 7, the mean (standard deviation [SD]) relative differences from the baseline in hematocrit in nonpregnant women were −0.70 (6.81) in the 3-day arm and 0.24 (6.27) in the 5-day arm (*P* = 0.32), and those in pregnant women were 4.5 (8.4) in the 3-day arm and −1.3 (8.6) in the 5-day arm (*P* = 0.08). By day 28, the mean (SD) relative differences in hematocrit in nonpregnant women were 0.70 (5.71) in the 3-day arm and 0.03 (6.06) in the 5-day arm (*P* = 0.96), and those in pregnant women were 6.9 (9.7) in the 3-day arm and 3.2 (11.6) in the 5-day arm (*P* = 0.16) (absolute hematocrit differences are shown in Table S2).

Median serum levels of aspartate aminotransferase (AST), alanine aminotransferase (ALT), creatinine (milligrams per deciliter), and albumin (grams per deciliter) were similar at baseline and at discharge between groups and treatment regimens (Table 3). The ALT level was above normal in 4 cases in the 5-day arm (18.2%; 4/22) versus 1 case (4.5%; 1/22) in the 3-day arm (*P* = 0.16) in the pregnant group and in 3 cases in the 5-day arm in the nonpregnant women. AST levels were above the normal range in 2 patients in the 5-day arm (2/21; 9.5%) and 3 patients in the 3-day arm (3/23; 13.0%) (*P* = 0.72) in the pregnant group and in 3 women (3/23; 13.0%) in the 5-day arm and 5 women (5/23; 21.7%) in the 3-day arm (*P* = 0.44) in the control group. None of the transaminase elevations were more than three times above the upper limit of normal, and all cases normalized within a month.

The serum creatinine levels were comparable and within the normal range between the two treatment arms at baseline and at discharge in pregnant and nonpregnant women. Creatinine was above the normal range in 5 cases (25.0%; 5/20) in the 5-day treatment arm and in 4 cases (28.6%; 4/14) in the 3-day treatment arm in the pregnant group (*P* = 0.82), but all values returned to normal within a month.

Artemether-lumefantrine was generally very well tolerated. At least one adverse event of mild or moderate intensity was reported in 65.6% of patients (63/96) (Table 5). All adverse events were classified as being unrelated to the study treatment. The most frequently reported symptoms were headache (*n* = 18), dizziness (*n* = 8), and gastrointestinal disorders (*n* = 8). Gastrointestinal disorders occurred more frequently in women who received 5-day AL than in those who received the 3-day treatment (*P* = 0.03).

**TABLE 5:** Adverse events by treatment arm and group

Adverse event(s)	No. (%) of women						*P* value
	3 days			5 days			
	Pregnant	Nonpregnant	All	Pregnant	Nonpregnant	All	
Headache	6	6	12 (25)	3	3	6 (12.5)	0.12
Gastrointestinal disorders	1	0	1 (2)	5	2	7 (14.6)	0.03
Dizziness	3	2	5 (10.4)	1	2	3 (6.3)	0.49
Influenza-like syndrome	3	0	3 (6.3)	0	1	1 (2)	0.31
Back pain	1	0	1 (2)	1	0	1 (2)	1
Epistaxis	1	0	1 (2)	1	0	1 (2)	1
High blood pressure	0	0	0 (0)	0	2	2 (4.2)	0.16
Decreased total WBCs	2	2	4 (8.3)	0	1	1 (2)	0.34
Decreased neutrophils	2	3	5 (10.4)	1	0	1 (2)	0.206
Increased ALT	1	0	1 (2)	3	3	6 (12.5)	0.23
Increased AST	3	5	8 (16.6)	2	3	5 (10.4)	0.55
Increased creatinine	4	3	7 (14.6)	5	4	9 (18.8)	0.60
Genital infection	0	0	0 (0)	2	0	2 (4.2)	0.16

Four serious adverse events, all in pregnant women, were reported, 3 in the 3-day arm and 1 in the 5-day arm. All were classified as being unrelated to the study treatment (described in the supplemental material).

### Electrocardiographic findings.

The electrocardiogram (ECG) values (QT corrected [QTc]; Fridericia and Bazett) were normal at admission in all patients, and none of the women had observed QTc intervals exceeding 500 ms during the trial.

The evaluation of the different correction factors by plotting QTc intervals versus heart rate showed a significant residual trend for both the Fridericia and the Bazett corrections (Fig. S1). Median peak concentrations were approximately 6,000 to 7,000 ng/ml in the four study arms (see Tables 7 and 8), resulting in a maximum Fridericia-corrected QT (QTcF) interval prolongation of 7.02 to 8.19 ms. A weak but significant (*P* = 0.0017) correlation was found between predicted lumefantrine concentrations and the Fridericia-corrected QT intervals (slope of 0.00117, corresponding to an increase in the QT of 1.17 ms with an increase in the lumefantrine concentration of 1,000 ng/ml) (Fig. 3A). No significant correlation was found between predicted lumefantrine concentrations and Bazett-corrected QT intervals (Fig. 3B).

### Delivery outcome and baby follow-up.

Forty-five women gave birth to a live baby: 41 women (85.4%) had a full-term pregnancy (between ≥37 and 40 weeks), and 4 (8.3%) had a preterm pregnancy (between ≥28 and <37 weeks). Three women (6.3%) had a stillbirth: two in the 3-day group and one in the 5-day group (Table 6; see also the description in the supplemental material). Four babies were small for gestational age in the 3-day group (12.5%), and 1 was small for gestational age in the 5-day group (4.17%; *P* = 0.61). A positive correlation between birth weight and BMI was observed (*P* = 0.032); the mothers of the babies with low birth weight (*n* = 7) had the lowest BMI values. There were no significant relationships between parasitemia, fever, or anemia and birth weights. One case of polydactylism (code Q69.9; ICD-10) of both hands was observed in a baby of a woman treated with the 5-day regimen at 31 weeks of gestation. This malformation was a family trait, and the exposure to the study drug occurred after the formation of fetal digits was complete; it was therefore classified as being unrelated to the study treatment. Two infant deaths at 3 months of age in the 5-day arm were attributed to severe diarrhea and suspected respiratory infection, respectively. These were also assessed as being unrelated to the study treatment. All other infants had normal physical and neurological development according to Denver developmental milestones (19) when assessed at 1 year of age.

**TABLE 6:** Delivery outcomes and adverse events in newborns and infants

Outcome parameter	Value		*P* value
	3 days	5 days	
No. of women analyzed	24	24	
Mean birth wt (g) (SD)	3,048.8 (526.0)	3,165.8 (469.8)	0.44
No. of low-birth-wt infants (<2,500 g) (%)	5 (22.7)	2 (9.1)	0.25
No. of premature infants (%)	3 (12.6)	1 (4.2)	0.36
No. of stillborn infants (%)	2 (8.3)	1 (4.2)	0.62
No. of boys (%)	10 (41.7)	12 (50)	0.58
No. of infants with congenital malformation (%)	0 (0)	1 (4.2)	1
Median newborn hematocrit (%) (range)	55 (43.8–65)	56.7 (33–74)	0.31
Median head circumference (cm) (range)	34.5 (31.0–37.0)	35.0 (31.5–36.0)	0.52
Median arm circumference (cm) (range)	11.0 (9.0–13.0)	11.8 (9.5–14.0)	0.65
Median infant length (cm) (range)	49.0 (43.0–55.0)	49.0 (46.0–54.0)	0.89
No. of cases of neonatal sepsis by clinical assessment	1	0	1
No. of infant deaths^*a*^	0	2	0.49

a The first death occurred at 16 weeks of age, and the second occurred at 7 months of age.

The median times between study treatment administration and delivery were not significantly different between women who received 3-day (70 days [IQR, 59 to 140 days]) and those who received 5-day (77 days [IQR, 50 to 129 days]) treatments (*P* = 0.672). There was no significant difference in the median times between study treatment and delivery between women without placental malaria (*n* = 35; 72 days [IQR, 51 to 129 days]) and those with placental malaria (*n* = 9; 68 days [IQR, 57 to 139 days]) (*P* = 0.673). However, some women might have had a second episode of malaria between the study treatment and delivery. All pregnant women in this study received intermittent preventive treatment with sulfadoxine-pyrimethamine (IPTp-SP), administered by the study nurses.

### Placenta histopathology.

Placental biopsy specimens were available from 44 patients. The remaining four patients delivered at home, and the placenta was discarded. Parasite-infected red blood cells (RBCs) were observed in 38.6% (17/44) of the placental biopsy specimens: 8 in the 3-day arm (*n* = 22) and 9 in the 5-day arm (*n* = 22). Eight women (47%) with a malaria-positive biopsy specimen also had a malaria-positive peripheral blood slide at delivery. There were more cases of active placental malaria in the 3-day arm (75%; *n* = 6/8) than in the 5-day arm (33.3%; *n* = 3/9), although the difference was not significant (*P* = 0.105). In one case (2.3%; 1/44), the mother had a positive peripheral blood smear at delivery, and malaria parasites were found in the cord blood (the baby was malaria negative). There was no significant difference in the median birth weights between those with and those without placental malaria: 3,300 g (range, 2,170 to 3,950 g) versus 3,070 g (range, 2,300 to 4,300 g) (*n* = 27; *P* = 0.85), respectively.

### Plasma lumefantrine levels on days 7 and 14.

The observed median (range) lumefantrine concentrations at day 7 in pregnant women were 597 ng/ml (216 to 928 ng/ml) in the 3-day arm and 1,545 ng/ml (537 to 3,650 ng/ml) in the 5-day arm (*P* < 0.001). In nonpregnant women, the median values were 541 ng/ml (315 to 1,780 ng/ml) in the 3-day arm and 1,995 ng/ml (457 to 4,270 ng/ml) in the 5-day arm (*P* < 0.001). Only one pregnant woman in the 3-day arm had a lumefantrine plasma level at day 7 below the 280-ng/ml cutoff. On day 14, the median (range) plasma lumefantrine levels in pregnant women were 151 ng/ml (68 to 265 ng/ml) in the 3-day arm and 247 ng/ml (99 to 541 ng/ml) in the 5-day arm (*P* < 0.001). In nonpregnant women, the median day 14 levels were 146 ng/ml (78 to 409 ng/ml) in the 3-day arm and 312 ng/ml (87 to 699 ng/ml) in the 5-day arm (*P* < 0.001).

### Pharmacokinetic properties of lumefantrine and desbutyl-lumefantrine.

**FIG 4:**
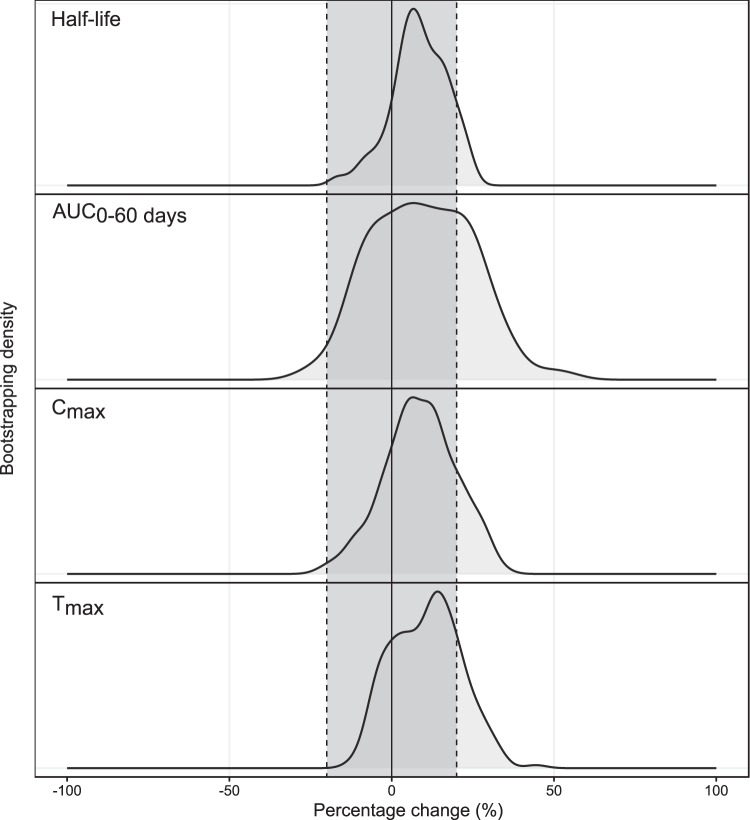
Density of secondary lumefantrine parameters based on a full covariate model investigating categorical pregnancy effects. The solid black line represents no covariate effect, and the dotted black lines represent a covariate effect of ±20%. T_max_ is the time to the maximum concentration, C_max_ is the maximum concentration, AUC is the total exposure, and half-life is the lumefantrine elimination half-life.

A total of 816 samples were analyzed for plasma lumefantrine concentrations, and 712 samples were analyzed for desbutyl-lumefantrine concentrations. A two-compartment disposition model was used to describe the pharmacokinetics of lumefantrine (Fig. S2). This was superior to other investigated disposition models (*P* < 0.05). A transit absorption model with four transit compartments performed better than all other absorption models (*P* < 0.05). Estimating the transit rate constant *k_tr_* and the absorption rate constant (*k_a_*) separately did not improve the model significantly, and they were assumed to be identical in the final model. None of the covariates evaluated (including body weight, pregnancy, and treatment duration) had a significant impact on the pharmacokinetic properties of lumefantrine. However, as a recent pooled analysis showed that body weight influences lumefantrine pharmacokinetic properties (20, 21), this was retained as a covariate in the final model. A full covariate approach evaluating secondary pharmacokinetic parameters showed no clinically significant impact of pregnancy on the exposure to lumefantrine (Fig. 4). Desbutyl-lumefantrine was modeled using a sequential approach. Concentration-time data were described by a three-compartment model, which was superior to all other disposition models (*P* < 0.05) (Fig. S2). Body weight was included in the model based on biological plausibility and improved the model fit. No other covariates were evaluated for the metabolite. Between-subject variability was estimated on all parameters. However, to stabilize the model, the variability in pharmacokinetic parameters associated with one peripheral compartment was fixed to zero. Final primary parameters, parameter precision, shrinkage, and secondary parameter estimates for the parent drug and metabolite are presented in Tables 7 and 8. Visual predictive checks (Fig. S2) of the final pharmacokinetic models showed a good description of the observed data. As expected, the overall parent drug and metabolite exposures were substantially higher after the extended 5-day regimen than after the standard 3-day regimen (Table 8).

**TABLE 7:** Final parameter estimates describing the population pharmacokinetics of lumefantrine and desbutyl-lumefantrine^*a*^

Drug and parameter estimate	Population estimate^*b*^ (RSE [%]^*c*^)	95% CI for population estimate^*c*^	BSV (RSE [%]^*c*^)	95% CI for BSV^*b*^	Shrinkage (%)^*b*^
Lumefantrine					
* F* (%)	1 fixed		45.1 (4.67)	40.3–49.3	5.49
No. of trans comp	4 fixed				
MTT (h)	6.33 (5.58)	5.59–7.06	51.7 (5.05)	46.4–57.7	9.29
CL/*F* (liters/h)	5.22 (4.18)	4.80–5.67			
* V*_c_/*F* (liters)	130 (7.20)	112–149	26.5 (6.76)	22.6–29.6	37.7
* Q*/*F* (liters/h)	1.68 (10.7)	1.22–1.72			
* V*_p_/*F* (liters)	144 (6.47)	134–170			
RUV	0.159 (6.19)	0.144–0.180			13.1

Desbutyl-lumefantrine					
CL/*F* (liters/h)	241 (3.68)	229–262	39.5 (7.89)	31.1–43.0	10.2
* V*_c_/*F* (liters)	2,570 (13.5)	2,190–3,640	181 (9.07)	156–321	13.1
* Q*_1_/*F* (liters/h)	189 (6.28)	165–211			
* V*_p1_/*F* (liters)	32,500 (11.2)	25,800–39,400			
* Q*_2_/*F* (liters/h)	4,340 (11.6)	2,860–4,440	67.6 (6.78)	60.6–84.8	33.4
* V*_p2_/*F* (liters)	12,800 (7.04)	11,300–14,900	63.7 (9.55)	57.9–88.1	18.4
RUV	0.0398 (3.61)	0.0350–0.0467			17.8

a Parameter estimates are presented for a typical 57-kg nonpregnant patient. CL/*F* is the apparent elimination clearance. *V*_c_/*F* is the apparent volume of distribution of the central compartment. *Q*/*F* is the intercompartmental clearance. *V*_p_/*F* is the apparent volume of distribution of the peripheral compartment. MTT is the mean transit time of absorption. RUV is the variance of the unexplained residual variability. No. of trans comp is the number of transit compartments used in the absorption model. F represents the relative bioavailability. BSV is the between-subject variability. Relative standard errors (RSE) were calculated as 100 × (standard deviation/mean value) for fixed effects and sigma and as 100 × (standard deviation/2 × mean value) for random effects presented as percent coefficients of variation (CV%). The 95% confidence intervals (CI) are given as the 2.5th to 97.5th percentiles of bootstrap estimates.

b Based on population mean values from NONMEM.

c Obtained from sampling importance resampling.

**TABLE 8:** Secondary parameters describing the population pharmacokinetics of lumefantrine and desbutyl-lumefantrine^*a*^

Drug and secondary parameter	Median value (range) for group			
	3 days		5 days	
	Nonpregnant	Pregnant	Nonpregnant	Pregnant
Lumefantrine				
* T*_max_ (h)	7.95 (4.81–15.2)	7.86 (4.47–16.7)	7.72 (5.54–11.7)	8.66 (4.76–14.0)
* C*_max_ (ng/ml)	6,170 (2,790–14,400)	6,290 (4,140–10,900)	7,320 (3,010–16,000)	6,600 (2,890–12,300)
AUC_0–60_ (h · μg/ml)	531 (267–1,250)	586 (321–887)	933 (374–2,030)	853 (381–1,650)
Half-life (days)	3.41 (3.22–3.69)	3.52 (3.22–3.87)	3.45 (3.24–3.92)	3.57 (3.33–3.97)
Day 7 concn (ng/ml)	541 (333–1,360)	665 (353–818)	1,900 (576–2,870)	1,280 (596–2,770)
* T_c_*_>280_ (days)	9.83 (7.63–14.5)	10.6 (6.98–12.5)	14.1 (9.03–17.8)	13.5 (9.63–17.3)

Desbutyl-lumefantrine				
* T*_max_ (h)	16.7 (9.0–29.9)	19.8 (10.1–32.1)	13.9 (7.39–29.6)	14.6 (8.11–24.9)
* C*_max_ (ng/ml)	42.7 (15.3–74.6)	51.4 (22.3–104)	56.2 (27.0–150)	75.5 (26.8–127)
AUC_0–60_ (h · μg/ml)	8.26 (3.09–15.9)	12.9 (6.41–24.5)	14.4 (5.98–41.8)	20.4 (10.4–35.5)
Half-life (days)	8.93 (7.41–10.9)	11.2 (8.15–16.0)	9.37 (7.75–12.1)	11.2 (9.39–13.7)

a *T*_max_ is the time to reach the maximum concentration (*C*_max_). AUC_0–60_ is the total exposure from the first dose to day 60.

### Pharmacokinetic properties of artemether and dihydroartemisinin.

**FIG 5:**
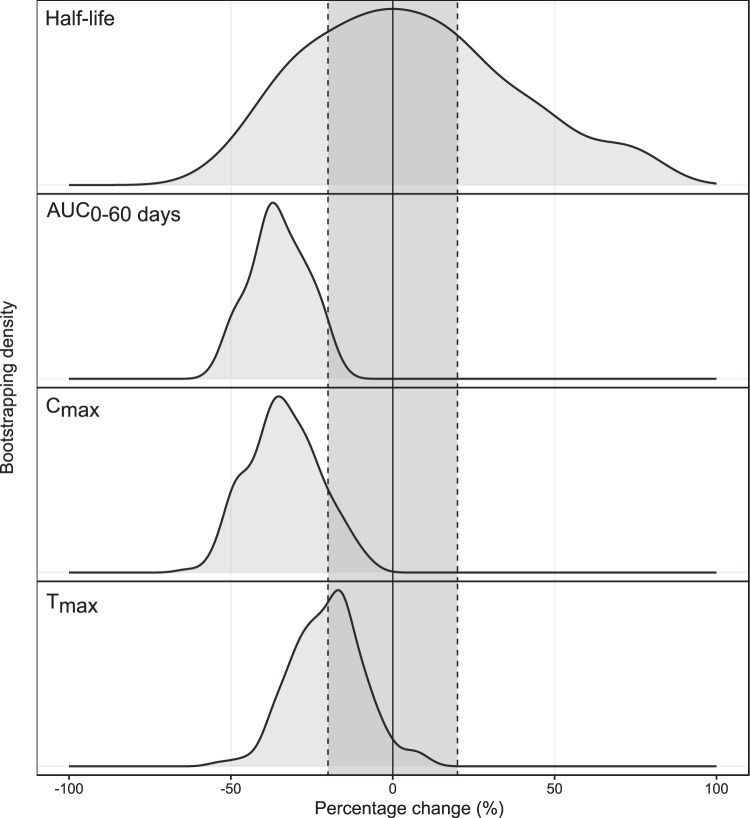
Density of secondary DHA parameters based on a full covariate model investigating categorical pregnancy effects. The solid black line represents no covariate effect, and the dotted black lines represent a covariate effect of ±20%. T_max_ is the time to the maximum concentration, C_max_ is the maximum concentration, AUC is the total exposure, and half-life is the DHA elimination half-life.

A total of 496 plasma samples were analyzed for artemether concentrations, and 488 samples were analyzed for DHA concentrations. A one-compartment disposition model was used to describe the pharmacokinetic properties of both artemether and DHA (Fig. S2). Two-compartment disposition models resulted in improved model fits for both AM and DHA (*P* < 0.05) but resulted in improbable parameter estimates (terminal elimination half-lives of 8.2 and 2,120 h, respectively). A transit compartment model with six transit compartments was superior to all other absorption models (*P* < 0.05). Estimating *k_tr_* and *k_a_* separately did not improve the model significantly, and they were assumed to be identical in the final model. Only 5.7% and 7.2% of artemether and DHA samples, respectively, were below the lower limit of quantification (LLOQ). Similar pharmacokinetic parameter estimates were obtained for both the one- and two-compartment disposition models when using the M1 or M3 method for LLOQ data (M1 and M3 described in the supplemental material). Thus, for parsimony, censored data (drug measurements below the LLOQ) were omitted in the final pharmacokinetic model (i.e., the M1 method). Estimated gestational age was a significant covariate affecting the relative artemether bioavailability, resulting in a 1.2% decrease in the relative bioavailability for each additional week in estimated gestational age. This resulted in a maximum difference of 48% between nonpregnant and pregnant women (i.e., by week 40). Estimating just a categorical difference between pregnant and nonpregnant women resulted in a 29.2% reduction in the relative bioavailability, while estimating a difference for each trimester resulted in a 25.2% reduction for trimester 2 and a 32.9% reduction for trimester 3 compared to nonpregnant women. The impact on DHA exposure was proportional to that established for artemether. No other covariates evaluated (including body weight and treatment duration) had a significant impact on the pharmacokinetic properties of artemether or DHA. However, body weight was retained in the model due to its strong biological prior, as argued above for lumefantrine. The full covariate approach, investigating the effect of pregnancy on secondary DHA pharmacokinetic parameters, showed significant reductions in exposure to artemether and DHA (Fig. 5). Final primary parameters, parameter precision, shrinkage, and secondary parameter estimates for artemether and DHA are presented in Tables 9 and 10. Goodness-of-fit plots (Fig. S4) and visual predictive checks (Fig. S3) of the final pharmacokinetic model showed a good description of the observed data. As expected, overall artemether and DHA exposures were substantially higher after the extended 5-day dosing than after the standard 3-day dosing (Table 10).

**TABLE 9:** Final parameter estimates describing the population pharmacokinetics of artemether and dihydroartemisinin^*a*^

Drug and parameter estimate	Population estimate^*b*^ (RSE [%]^*c*^)	95% CI for population estimate^*c*^	IIV/IOV^*b*^ (RSE [%]^*c*^)	95% CI for IIV/IOV	Shrinkage (%)^*b*^
Artemether					
* F* (%)	1 fixed		36.5 (7.51)	30.0 to 41.7	25.0
No. of trans comp	6 fixed				
MTT (h)	0.487 (10.2)	0.386 to 0.586	67.3 (8.82)	53.7 to 80.5	49.6
CL/*F* (liters/h)	365 (5.72)	325 to 408	20.5 (7.60)	16.9 to 23.1	33.0
* V*_c_/*F* (liters)	1,350 (6.58)	1,190 to 1,530	17.5 (12.7)	14.4 to 22.4	50.7
RUV	0.549 (7.05)	0.485 to 0.629			8.04
EGA on *F* (%)	−0.175 (9.07)	−0.202 to −0.144			

Dihydroartemisinin					
CL/*F* (liters/h)	363 (5.44)	314 to 400	25.6 (15.6)	20.2 to 35.3	44.7
* V*_c_/*F* (liters)	110 (12.7)	72.8 to 133			
RUV	0.474 (7.75)	0.211 to 0.315			10.5

a Parameter estimates are presented for a 57-kg nonpregnant patient. CL/*F* is the apparent elimination clearance. *V*_c_/*F* is the apparent volume of distribution of the central compartment. MTT is the mean transit time of the absorption phase. RUV is the variance of the unexplained residual variability. No. of trans comp is the number of transit compartments used in the absorption model. Percent coefficients of variation (CV%) for interindividual variability (IIV) and interoccasion variability (IOV) were calculated as $\sqrt{e^{{\text{ω}}^{2}}-1}$. Relative standard errors (RSE) were calculated as 100 × (standard deviation/mean value) for fixed effects and sigma and as 100 × (standard deviation/2 × mean value) for random effects presented as CV%. The 95% confidence intervals (CI) are given as the 2.5th to 97.5th percentiles of bootstrap estimates.

b Based on population mean values from NONMEM.

c Obtained from sampling importance resampling.

**TABLE 10:** Secondary parameters describing the population pharmacokinetics of artemether and dihydroartemisinin^*a*^

Drug and secondary parameter	Median value (range) for group			
	3 days		5 days	
	Nonpregnant	Pregnant	Nonpregnant	Pregnant
Artemether				
* T*_max_ (h)	0.93 (0.71–2.41)	0.890 (0.540–1.83)	0.880 (0.710–1.71)	0.880 (0.530–1.63)
* C*_max_ (ng/ml)	67.6 (30.1–136)	42.5 (25.3–86.8)	63.3 (35.1–137)	42.5 (29.0–67.7)
AUC_0–60_ (h · ng/ml)	1,710 (617–3,870)	948 (511–2,530)	2,120 (1,210–4,110)	1,610 (929–2,370)
Half-life (h)	2.67 (2.09–4.71)	2.53 (1.73–4.05)	2.39 (1.59–2.98)	2.45 (1.73–3.78)

Dihydroartemisinin				
* T*_max_ (h)	1.38 (1.06–2.71)	1.35 (1.10–2.16)	1.35 (1.15–2.23)	1.35 (1.05–1.96)
* C*_max_ (ng/ml)	57.3 (24.7–114)	42.5 (25.3–86.8)	59.8 (28.1–137)	38.0 (21.8–65.6)
AUC_0–60_ (h · ng/ml)	1,600 (558–2,700)	948 (511–2,530)	2,350 (1,260–4,740)	1,500 (932–2,380)
Half-life (h)	0.210 (0.140–0.300)	0.210 (0.130–0.300)	0.220 (0.180–0.290)	0.210 (0.170–0.270)

a *T*_max_ is the time to reach the maximum concentration (*C*_max_). AUC_0–60_ is the total exposure from the first dose to day 60.

## DISCUSSION

Artemether-lumefantrine is the most widely used artemisinin-based combination treatment (ACT) worldwide for the treatment of uncomplicated malaria in pregnancy. Previous studies suggested that exposure to these drugs is reduced in pregnancy (5, 9, 11, 22, 23). Low drug levels and high parasite burdens increase the risk of treatment failure and are conducive to *de novo* resistance selection (8, 13). A reassessment of ACT dosing in pregnancy would ensure the highest possible efficacy of malaria treatments in pregnant women. Pharmacokinetic modeling suggested that a twice-daily extended artemether-lumefantrine regimen given for 5 days would be preferable to the current standard 3-day dosing regimen. Artemether-lumefantrine is an extremely well-tolerated and safe antimalarial, but most of these reassuring data are derived from the standard regimen.

In this prospective randomized study, the tolerability and safety of the extended regimen were excellent, with no evidence of drug-related toxicity. There were no adverse effects in the pregnant woman, nor were any significant electrocardiographic, biochemical, or hematological changes detected. A weak correlation was found between predicted lumefantrine concentrations and the Fridericia-corrected QT, showing an increase of 1.17 ms with an increase in the lumefantrine concentration of 1,000 ng/ml, resulting in a median QTcF prolongation of <10 ms in all treatment groups. Birth and infant (up to the age of 1 year) outcomes did not differ significantly between the two regimens, both of which achieved 100% cure rates in pregnant and nonpregnant women. This high cure rate in western Democratic Republic of the Congo (DRC) was observed 2 years after the introduction of artemether-lumefantrine as a second first-line treatment (in addition to amodiaquine-artesunate) for uncomplicated malaria as part of the revised National Guidelines of Treatment of Malaria (DRC National Malaria Control Program, 2012). The parasite clearance times were significantly shorter in pregnant women (3.30 h [range, 1.39 to 7.83 h]) than in nonpregnant women (2.43 h [1.05 to 6.00 h]). This was not associated with the early exposures to artemether and DHA and suggests that pregnancy may reduce splenic clearance function. Malaria parasites were detected in the placentas of 38% of women. In the study area, malaria transmission is high and perennial, so it is likely that these placental infections resulted from prior or subsequent malaria episodes, not treated during the study, which remained subpatent, and/or reduced efficacy of sulfadoxine-pyrimethamine used as a preventive treatment. Pregnant women had lower exposures to both artemether and DHA than nonpregnant women, resulting in 1.2% decreased exposure per week increase in gestational age. By term, these exposures were reduced by 48% compared to nonpregnant patients. This finding was also confirmed with a full covariate model, which showed that exposure to dihydroartemisinin was significantly reduced in pregnant women. These results are comparable with previous findings in pregnant Congolese women, in the same study area, treated with artesunate (24, 25). Lower levels of artemether and DHA were also reported in Karen pregnant women (9, 10, 22, 26) and Tanzanian pregnant women treated with artemether-lumefantrine (27).

In the present study, the results of the nonlinear mixed-effects modeling showed that pregnancy did not affect significantly any of the pharmacokinetic parameters for either lumefantrine or the desbutyl metabolite. The overall exposure, as expected, was substantially improved in the 5-day regimen (which consisted of an increase of 40% of the total dose). The observed median day 7 lumefantrine plasma concentrations were not different between pregnant and nonpregnant women. The median levels in our study (596.5 ng/ml [range, 216 to 928 ng/ml]) were comparable to those found in Uganda (488 ng/ml [30.7 to 3,550 ng/ml]) (11) after the 3-day treatment, with only one patient with a level below 280 ng/ml (the cutoff which best correlates with treatment outcomes). In nonpregnant Congolese women, however, the day 7 values were lower (541 ng/ml [315 to 1,780 ng/ml]) than in Ugandan nonpregnant women (720 ng/ml [339 to 2,150 ng/ml]). In the extended-regimen arm, as expected, the day 7 levels were substantially higher: 1,545 ng/ml (537 to 3,650 ng/ml) in pregnant women and 1,995 ng/ml (457 to 4,270 ng/ml) in nonpregnant women.

These results contrast with findings from previous studies in Asia and Africa investigating the pharmacokinetic properties of lumefantrine in pregnant women. In Asia, a reduced exposure (AUC) to lumefantrine was observed in pregnant Karen women (using a noncompartmental analysis), 38% of whom had day 7 lumefantrine venous blood concentrations of <280 ng/ml. This was associated with increased lumefantrine elimination (22). In a larger study in the same area (9), 35% of pregnant women had day 7 lumefantrine capillary blood concentrations (observed) below 355 ng/ml (corresponding to 280 ng/ml venous blood), which were associated with poor treatment outcomes (81%; day 42 PCR corrected). A population pharmacokinetic study nested in the above-mentioned clinical trial (10) estimated a 7.2% increase in the volume of distribution by week of gestational age and 64% higher odds for recrudescence with each 100-ng/ml decrease in the day 7 lumefantrine concentration. In Uganda, where the rate of transmission of malaria is high, 32% of pregnant women (11) had day 7 lumefantrine venous blood concentrations (observed) of <280 ng/ml. Cure rates were very high, but lower lumefantrine levels were associated with the reappearance of malaria in the follow-up period (presumed earlier reinfections). A noncompartmental pharmacokinetic analysis of these data (28) found no statistical difference in lumefantrine exposures between pregnant and nonpregnant women, nor was there a significant correlation between estimated gestational age and lumefantrine exposures. A population pharmacokinetic study nested in the main clinical trial (26), using additional day 7 capillary plasma levels, estimated that pregnancy had a significant impact on lumefantrine pharmacokinetics, with day 7 concentrations that were 27% lower than those in nonpregnant women, associated with a 36.5% decrease in the intercompartmental clearance. Artemether-lumefantrine antimalarial efficacy in pregnancy is significantly lower on the Thailand-Myanmar border. Differences in efficacy could result from host-specific differences such as BMI and host immunity (both of which are much lower in Asia) and reduced parasite susceptibility in Southeast Asia. A lower relative bioavailability of lumefantrine was also observed with a population pharmacokinetic modeling approach in Tanzanian pregnant women than in nonpregnant women treated with artemether-lumefantrine (23). In another study in Uganda, no differences were found in exposures to artemether, DHA, and lumefantrine between pregnant and nonpregnant subjects, although a shorter terminal half-life of lumefantrine was observed in pregnant women (29).

The present study was not powered to detect low frequencies of adverse events, and by study design, women in the 5-day arm were observed for a longer period than were those in the 3-day arm. However, the drug tolerability and safety results in this small cohort are reassuring. Longer courses risk reduced adherence. In this study, the treatment was administered under direct observation and with milk to maximize drug absorption. In daily clinical practice, adherence to a 5-day treatment and coadministration with fatty foods might be suboptimal. A new solid-dispersion formulation of lumefantrine is currently under development by Novartis (30). The preliminary results showed a significant improvement in drug bioavailability in healthy subjects. In the future, this new formulation could contribute to a better efficacy of artemether-lumefantrine in clinical practice.

### Conclusions.

In conclusion, both the current and extended regimens of artemether-lumefantrine were highly efficacious, well tolerated, and safe in Congolese pregnant women with uncomplicated falciparum malaria. The 5-day treatment increases drug exposures without apparent toxicity and is therefore a promising option for the treatment of malaria in pregnancy in areas where efficacy has declined.

## MATERIALS AND METHODS

### Study site.

This study was conducted by the Kinshasa School of Public Health-University of Oxford Medical Research Unit, Kinshasa, Democratic Republic of the Congo, at the Maternity Hospital of Kingasani, located in a suburban area of Kinshasa. Malaria transmission is high and perennial in this region.

### Trial design and procedures.

This was an open-label, two-arm, individually randomized controlled clinical trial. Women aged ≥18 to ≤45 years were considered eligible if they had uncomplicated falciparum malaria defined as a positive blood slide with asexual Plasmodium falciparum parasitemia of between 100 and 200,000 parasites/μl and a hematocrit of ≥21%. Pregnant women were included if the estimated gestational age was ≥14 weeks and they had a singleton viable fetus confirmed by ultrasound using Hadlock’s method (31). Women were excluded if they had severe malaria, a medical condition requiring concomitant drug treatment or transfer to a different hospital, or HIV-positive status or if they reported an intake of artemether-lumefantrine within the two previous weeks, a known allergy to the study drug, previous participation in the current trial, or participation in other studies. Pregnant women with signs of labor or fetal abnormalities identified by ultrasound were also excluded. The study was explained in the local language (French or Lingala), and informed consent was signed before inclusion. All patients were hospitalized during and immediately after drug administration. Totals of 24 women in the 2nd trimester, 24 women in the 3rd trimester, and 48 nonpregnant women were to be recruited.

### Drug regimens.

Patients were randomly allocated to a 3-day or 5-day artemether-lumefantrine regimen (Coartem 20/120-mg tablets; Novartis, Basel, Switzerland) according to their status (2nd trimester, 3rd trimester, or nonpregnant). The randomization lists were computer generated by an independent statistician, with sampling time points randomized within each of the 6 blocks.

In the 3-day regimen, patients received 6 doses (at 0, 8, 24, 36, 48, and 60 h), and in the 5-day regimen, patients received 10 doses (at 0, 8, 24, 36, 48, 60, 72, 84, 96, and 108 h). Each dose (4 tablets; 80 mg of artemether and 480 mg of lumefantrine) was administered under direct observation with 200 ml of milk. If vomiting occurred within 30 min, a full dose was repeated, and if vomiting occurred between 30 min and 1 h, half the dose was repeated. Recurrent episodes of malaria were to be treated with oral quinine sulfate (10 mg salt/kg of body weight), three times daily for 7 days. All patients were admitted for the duration of the treatment, and doses were supervised.

### Outcome measurements.

The outcomes were the therapeutic efficacy (32), physical and neurological development during the first year of life of children born to women enrolled in the study (19), the occurrence and frequency of adverse events, and characterization of drug plasma concentration profiles (pharmacokinetics) of artemether and lumefantrine and their active metabolites dihydroartemisinin and desbutyl-lumefantrine, respectively.

### Clinical and laboratory methods.

Data on clinical symptoms were collected daily during hospitalization (i.e., 3 days in one group and 5 days in the other group) and then weekly until day 42 or at delivery, whichever occurred first. Babies were assessed at delivery and at 1, 3, 6, and 12 months according to Denver developmental milestones (19).

Hemoglobin was measured from capillary blood at screening using Hemocue Hb301 (Angelholm, Sweden). Hematocrit was measured at screening, at baseline, every 12 h during hospitalization, and weekly using a Hawksley Haematospin 1400 instrument (Hawksley & Sons, Ltd., United Kingdom). Total and differential white blood cell counts were performed by Sysmex at baseline and discharge. Plasma alanine aminotransferase (ALT), aspartate aminotransferase (AST), albumin, and creatinine levels were measured by a SEAC Screen Master from blood collected in lithium heparin tubes at baseline and 48 h in the 3-day group and 96 h in the 5-day group. Blood films were prepared and read using standard procedures at screening; at 0, 6, and 12 h; and then every 12 h until two negative blood smears were observed. Blood samples from recurrent episodes of malaria during follow-up and at delivery from peripheral blood in the mother and from cord blood and heel prick in the newborn were analyzed by PCR to distinguish recrudescence from new infections using a standard parasite genotyping methodology (33).

A 12-lead electrocardiogram was recorded before or within 28 h after the first dose and at discharge (3 or 5 days according to treatment group, 64.3 to 131 h after the first dose) using a Cardiofax ECG-9620 electrocardiograph (Nihon Kohden Corporation, Japan), which provides automated Fridericia-corrected QT intervals (QTcF). The instrument-generated QT and QTcF values were used to calculate Bazett-corrected QT (QTcB) values. The correlation between QT, QTcF, and QTcB and heart rates was evaluated with unweighted ordinary linear regression using GraphPad Prism 8.2.0.

Venous blood for drug measurement was drawn into prechilled lithium heparin tubes at 3 fixed time points (0, 7, and 14 days) and taken at random 7 times within the following time windows, after the first drug intake (hour zero): 0 to 3 h, 3 to 6 h, 6 to 12 h, 12 to 60 h, 60 to 72 h, 72 to 144 h, and 192 to 336 h. Blood samples were centrifuged at 1,400 × *g* for 7 min at +4°C, and plasma was then stored in cryovials at −80°C until shipment on dry ice to the Department of Clinical Pharmacology at the Mahidol-Oxford Research Unit in Thailand.

A placenta biopsy was performed within 4 h of delivery, and the specimen was fixed in 10% buffered formalin before routine processing and hematoxylin and eosin (H&E) staining for microscopic examination to determine the presence of active placental malaria and any other pathology.

Safety outcomes were adverse events as defined by International Council for Harmonisation good clinical practice guidelines (34).

### Drug assays.

Artemether and DHA plasma concentrations were measured by liquid chromatography-tandem mass spectrometry (LC-MS/MS) (35). Quality control for drug quantification was done by comparing the samples to three quality control samples at concentrations of 3.46 ng/ml, 36 ng/ml, and 375 ng/ml, analyzed in triplicates within each batch of clinical study samples. The lower limit of detection (LOD) was 0.5 ng/ml, and the lower limit of quantification (LLOQ) was set to 1.4 ng/ml for both compounds. Lumefantrine and desbutyl-lumefantrine plasma concentrations were also measured by LC-MS/MS, adapted from a previously reported high-performance liquid chromatography (HPLC) method (36, 37). Triplicates of quality control samples were analyzed at concentrations of 33.5 ng/ml, 709 ng/ml, and 150,000 ng/ml. The LOD was 2.5 ng/ml, and the LLOQ was set to 9.71 ng/ml.

### Statistical analysis.

For the per-protocol analysis, a chi-squared test, a *z*-test, and Fisher’s exact test were used to compare proportions. Analysis of variance (ANOVA) was used for normally distributed continuous data, and a Kruskal-Wallis test was used for continuous data with a skewed distribution. Paired *t* tests were used for comparisons within the same patient over time. All statistical analyses were performed using STATA IC software (release 14.0; STATA Corporation, College Station, Texas, USA) or GraphPad Prism 8.2.0. The parasite clearance half-life (PC_1/2_) was estimated using the WorldWide Antimalarial Resistance Network (WWARN) parasite clearance estimator, modified to allow for a lower threshold of parasitemia at time zero (38).

### Pharmacokinetic analysis.

Population-based nonlinear mixed-effects modeling was performed using NONMEM v7.3 software (Icon Development Solutions, Ellicott City, MD, USA) (see the supplemental material).

### Ethical approval.

The study was approved by the Oxford Tropical Research Ethics Committee, the Kinshasa School of Public Health Institutional Review Board, and the Ministry of Public Health of DRC. The study was conducted in accordance with good clinical practice guidelines (ClinicalTrials.gov identifier NCT01916954).

### Data availability.

The data that support the findings are available from the authors upon reasonable request and with permission of the University of Oxford and the Kinshasa School of Public Health.

## Supplementary Material

Supplemental file 1

## References

[1] White NJ, McGready RM, Nosten FH 2008 New medicines for tropical diseases in pregnancy: catch-22. PLoS Med 5:e133. doi:10.1371/journal.pmed.0050133. 18563964 PMC2429948

[2] Anderson GD 2005 Pregnancy-induced changes in pharmacokinetics: a mechanistic-based approach. Clin Pharmacokinet 44:989–1008. doi:10.2165/00003088-200544100-00001. 16176115

[3] Hodge LS, Tracy TS 2007 Alterations in drug disposition during pregnancy: implications for drug therapy. Expert Opin Drug Metab Toxicol 3:557–571. doi:10.1517/17425225.3.4.557. 17696806

[4] Jeong H 2010 Altered drug metabolism during pregnancy: hormonal regulation of drug-metabolizing enzymes. Expert Opin Drug Metab Toxicol 6:689–699. doi:10.1517/17425251003677755. 20367533 PMC3686288

[5] McGready R, Stepniewska K, Lindegardh N, Ashley EA, La Y, Singhasivanon P, White NJ, Nosten F 2006 The pharmacokinetics of artemether and lumefantrine in pregnant women with uncomplicated falciparum malaria. Eur J Clin Pharmacol 62:1021–1031. doi:10.1007/s00228-006-0199-7. 17053895

[6] Kloprogge F, McGready R, Hanpithakpong W, Blessborn D, Day NP, White NJ, Nosten F, Tarning J 2015 Lumefantrine and desbutyl-lumefantrine population pharmacokinetic-pharmacodynamic relationships in pregnant women with uncomplicated Plasmodium falciparum malaria on the Thailand-Myanmar border. Antimicrob Agents Chemother 59:6375–6384. doi:10.1128/AAC.00267-15. 26239986 PMC4576090

[7] Tarning J, Kloprogge F, Piola P, Dhorda M, Muwanga S, Turyakira E, Nuengchamnong N, Nosten F, Day NP, White NJ, Guerin PJ, Lindegardh N 2012 Population pharmacokinetics of artemether and dihydroartemisinin in pregnant women with uncomplicated Plasmodium falciparum malaria in Uganda. Malar J 11:293. doi:10.1186/1475-2875-11-293. 22913677 PMC3502166

[8] White NJ, van Vugt M, Ezzet F 1999 Clinical pharmacokinetics and pharmacodynamics of artemether-lumefantrine. Clin Pharmacokinet 37:105–125. doi:10.2165/00003088-199937020-00002. 10496300

[9] McGready R, Tan SO, Ashley EA, Pimanpanarak M, Viladpai-Nguen J, Phaiphun L, Wustefeld K, Barends M, Laochan N, Keereecharoen L, Lindegardh N, Singhasivanon P, White NJ, Nosten F 2008 A randomised controlled trial of artemether-lumefantrine versus artesunate for uncomplicated Plasmodium falciparum treatment in pregnancy. PLoS Med 5:e253. doi:10.1371/journal.pmed.0050253. 19265453 PMC2605900

[10] Tarning J, McGready R, Lindegardh N, Ashley EA, Pimanpanarak M, Kamanikom B, Annerberg A, Day NP, Stepniewska K, Singhasivanon P, White NJ, Nosten F 2009 Population pharmacokinetics of lumefantrine in pregnant women treated with artemether-lumefantrine for uncomplicated Plasmodium falciparum malaria. Antimicrob Agents Chemother 53:3837–3846. doi:10.1128/AAC.00195-09. 19564366 PMC2737887

[11] Piola P, Nabasumba C, Turyakira E, Dhorda M, Lindegardh N, Nyehangane D, Snounou G, Ashley EA, McGready R, Nosten F, Guerin PJ 2010 Efficacy and safety of artemether-lumefantrine compared with quinine in pregnant women with uncomplicated Plasmodium falciparum malaria: an open-label, randomised, non-inferiority trial. Lancet Infect Dis 10:762–769. doi:10.1016/S1473-3099(10)70202-4. 20932805

[12] Barnes KI, Watkins WM, White NJ 2008 Antimalarial dosing regimens and drug resistance. Trends Parasitol 24:127–134. doi:10.1016/j.pt.2007.11.008. 18262470

[13] White NJ, Pongtavornpinyo W, Maude RJ, Saralamba S, Aguas R, Stepniewska K, Lee SJ, Dondorp AM, White LJ, Day NP 2009 Hyperparasitaemia and low dosing are an important source of anti-malarial drug resistance. Malar J 8:253. doi:10.1186/1475-2875-8-253. 19906307 PMC2784792

[14] Lefèvre G, Thomsen M 1999 Clinical pharmacokinetics of artemether and lumefantrine (Riamet). Clin Drug Invest 18:467–480. doi:10.2165/00044011-199918060-00006.

[15] Navaratnam V, Mansor SM, Sit NW, Grace J, Li Q, Olliaro P 2000 Pharmacokinetics of artemisinin-type compounds. Clin Pharmacokinet 39:255–270. doi:10.2165/00003088-200039040-00002. 11069212

[16] Ezzet F, Mull R, Karbwang J 1998 Population pharmacokinetics and therapeutic response of CGP 56697 (artemether + benflumetol) in malaria patients. Br J Clin Pharmacol 46:553–561. doi:10.1046/j.1365-2125.1998.00830.x. 9862244 PMC1873796

[17] Wong RP, Salman S, Ilett KF, Siba PM, Mueller I, Davis TM 2011 Desbutyl-lumefantrine is a metabolite of lumefantrine with potent in vitro antimalarial activity that may influence artemether-lumefantrine treatment outcome. Antimicrob Agents Chemother 55:1194–1198. doi:10.1128/AAC.01312-10. 21199927 PMC3067122

[18] Ashley EA, Stepniewska K, Lindegardh N, McGready R, Annerberg A, Hutagalung R, Singtoroj T, Hla G, Brockman A, Proux S, Wilahphaingern J, Singhasivanon P, White NJ, Nosten F 2007 Pharmacokinetic study of artemether-lumefantrine given once daily for the treatment of uncomplicated multidrug-resistant falciparum malaria. Trop Med Int Health 12:201–208. doi:10.1111/j.1365-3156.2006.01785.x. 17300626

[19] Frankenburg WK, Dodds J, Archer P, Shapiro H, Bresnick B 1992 The Denver II: a major revision and restandardization of the Denver developmental screening test. Pediatrics 89:91–97. 1370185

[20] Kloprogge F, Workman L, Borrmann S, Tekete M, Lefevre G, Hamed K, Piola P, Ursing J, Kofoed PE, Martensson A, Ngasala B, Bjorkman A, Ashton M, Friberg Hietala S, Aweeka F, Parikh S, Mwai L, Davis TME, Karunajeewa H, Salman S, Checchi F, Fogg C, Newton PN, Mayxay M, Deloron P, Faucher JF, Nosten F, Ashley EA, McGready R, van Vugt M, Proux S, Price RN, Karbwang J, Ezzet F, Bakshi R, Stepniewska K, White NJ, Guerin PJ, Barnes KI, Tarning J 2018 Artemether-lumefantrine dosing for malaria treatment in young children and pregnant women: a pharmacokinetic-pharmacodynamic meta-analysis. PLoS Med 15:e1002579. doi:10.1371/journal.pmed.1002579. 29894518 PMC5997317

[21] Worldwide Antimalarial Resistance Network AL Dose Impact Study Group. 2015 The effect of dose on the antimalarial efficacy of artemether-lumefantrine: a systematic review and pooled analysis of individual patient data. Lancet Infect Dis 15:692–702. doi:10.1016/S1473-3099(15)70024-1. 25788162 PMC4632191

[22] McGready R, Stepniewska K, Ward SA, Cho T, Gilveray G, Looareesuwan S, White NJ, Nosten F 2006 Pharmacokinetics of dihydroartemisinin following oral artesunate treatment of pregnant women with acute uncomplicated falciparum malaria. Eur J Clin Pharmacol 62:367–371. doi:10.1007/s00228-006-0118-y. 16552504

[23] Mosha D, Guidi M, Mwingira F, Abdulla S, Mercier T, Decosterd LA, Csajka C, Genton B 2014 Population pharmacokinetics and clinical response for artemether-lumefantrine in pregnant and nonpregnant women with uncomplicated Plasmodium falciparum malaria in Tanzania. Antimicrob Agents Chemother 58:4583–4592. doi:10.1128/AAC.02595-14. 24867986 PMC4136066

[24] Onyamboko MA, Meshnick SR, Fleckenstein L, Koch MA, Atibu J, Lokomba V, Douoguih M, Hemingway-Foday J, Wesche D, Ryder RW, Bose C, Wright LL, Tshefu AK, Capparelli EV 2011 Pharmacokinetics and pharmacodynamics of artesunate and dihydroartemisinin following oral treatment in pregnant women with asymptomatic Plasmodium falciparum infections in Kinshasa DRC. Malar J 10:49. doi:10.1186/1475-2875-10-49. 21352601 PMC3056842

[25] Morris CA, Duparc S, Borghini-Fuhrer I, Jung D, Shin CS, Fleckenstein L 2011 Review of the clinical pharmacokinetics of artesunate and its active metabolite dihydroartemisinin following intravenous, intramuscular, oral or rectal administration. Malar J 10:263. doi:10.1186/1475-2875-10-263. 21914160 PMC3180444

[26] Kloprogge F, Piola P, Dhorda M, Muwanga S, Turyakira E, Apinan S, Lindegardh N, Nosten F, Day NP, White NJ, Guerin PJ, Tarning J 2013 Population pharmacokinetics of lumefantrine in pregnant and nonpregnant women with uncomplicated Plasmodium falciparum malaria in Uganda. CPT Pharmacometrics Syst Pharmacol 2:e83. doi:10.1038/psp.2013.59. 24226803 PMC3852159

[27] Tarning J, Rijken MJ, McGready R, Phyo AP, Hanpithakpong W, Day NP, White NJ, Nosten F, Lindegardh N 2012 Population pharmacokinetics of dihydroartemisinin and piperaquine in pregnant and nonpregnant women with uncomplicated malaria. Antimicrob Agents Chemother 56:1997–2007. doi:10.1128/AAC.05756-11. 22252822 PMC3318332

[28] Tarning J, Kloprogge F, Dhorda M, Jullien V, Nosten F, White NJ, Guerin PJ, Piola P 2013 Pharmacokinetic properties of artemether, dihydroartemisinin, lumefantrine, and quinine in pregnant women with uncomplicated Plasmodium falciparum malaria in Uganda. Antimicrob Agents Chemother 57:5096–5103. doi:10.1128/AAC.00683-13. 23917320 PMC3811434

[29] Nyunt MM, Nguyen VK, Kajubi R, Huang L, Ssebuliba J, Kiconco S, Mwima MW, Achan J, Aweeka F, Parikh S, Mwebaza N 2015 Artemether-lumefantrine pharmacokinetics and clinical response are minimally altered in pregnant Ugandan women treated for uncomplicated falciparum malaria. Antimicrob Agents Chemother 60:1274–1282. doi:10.1128/AAC.01605-15. 26666942 PMC4775973

[30] Jain JP, Leong FJ, Chen L, Kalluri S, Koradia V, Stein DS, Wolf MC, Sunkara G, Kota J 2017 Bioavailability of lumefantrine is significantly enhanced with a novel formulation approach, an outcome from a randomized, open-label pharmacokinetic study in healthy volunteers. Antimicrob Agents Chemother 61:e00868-17. doi:10.1128/AAC.00868-17. 28630183 PMC5571342

[31] Hadlock FP, Deter RL, Carpenter RJ, Park SK 1981 Estimating fetal age: effect of head shape on BPD. AJR Am J Roentgenol 137:83–85. doi:10.2214/ajr.137.1.83. 6787895

[32] WHO. 2009 Methods for surveillance of antimalarial drug efficacy. WHO, Geneva, Switzerland.

[33] WHO. 2007 Methods and techniques for clinical trials on antimalarial drug efficacy: genotyping to identify parasite populations. Informal consultation organized by the Medicines for Malaria Venture and cosponsored by the World Health Organization, 29–31 May 2007, Amsterdam, The Netherlands. WHO, Geneva, Switzerland.

[34] ICH Expert Working Group. 1996 ICH harmonised tripartite guideline for good clinical practice E6(R1). International Council for Harmonisation, Geneva, Switzerland.

[35] Hanpithakpong W, Kamanikom B, Singhasivanon P, White NJ, Day NP, Lindegardh N 2009 A liquid chromatographic-tandem mass spectrometric method for determination of artemether and its metabolite dihydroartemisinin in human plasma. Bioanalysis 1:37–46. doi:10.4155/bio.09.6. 21083186

[36] Lindegardh N, Tarning J, Toi PV, Hien TT, Farrar J, Singhasivanon P, White NJ, Ashton M, Day NP 2009 Quantification of artemisinin in human plasma using liquid chromatography coupled to tandem mass spectrometry. J Pharm Biomed Anal 49:768–773. doi:10.1016/j.jpba.2008.12.014. 19162422 PMC2658735

[37] Annerberg A, Singtoroj T, Tipmanee P, White NJ, Day NP, Lindegardh N 2005 High throughput assay for the determination of lumefantrine in plasma. J Chromatogr B Analyt Technol Biomed Life Sci 822:330–333. doi:10.1016/j.jchromb.2005.06.022. 16005694

[38] Flegg JA, Guerin PJ, White NJ, Stepniewska K 2011 Standardizing the measurement of parasite clearance in falciparum malaria: the parasite clearance estimator. Malar J 10:339. doi:10.1186/1475-2875-10-339. 22074219 PMC3305913

