# Supplementary material for: A Randomized Controlled Trial of Three- versus Five-Day Artemether-Lumefantrine Regimens for Treatment of Uncomplicated Plasmodium falciparum Malaria in Pregnancy in Africa
Source: Antimicrob Agents Chemother. 2020 Feb 21;64(3):e01140-19. doi: 10.1128/AAC.01140-19 (PMC7038309; doi:10.1128/AAC.01140-19)
Supplement: Supplemental file 1 [file AAC.01140-19-s0001.pdf]

## Supplementary material

### Serious Adverse Events in pregnant women

1) The subject (20 years 8 months old) was enrolled in the 3-day artemether-lumefantrine arm (*P. falciparum* 240/µL; Hb 9.3; EGA at enrolment 33 weeks). The SAE occurred eight weeks after enrolment. The patient delivered at home a baby in breech presentation with the retention of the head in the vagina canal. By the time she was brought to hospital the baby was deceased. The event was classified as not related to the study drug.
2) The subject (21 years 11 months old) was enrolled in the 3-day artemether-lumefantrine arm (*P. falciparum* 3642/µL; Hb 9.9; EGA at enrolment 28 weeks). The SAE occurred ten weeks after enrolment. Two weeks before delivery, the mother reported fever and headache for which she took paracetamol at home as self-medication. The day of delivery there was no audible foetal heart beat and she delivered a macerated stillborn. The event was classified as not related to the study drug and the alternative aetiology was a suspected infection.
3) The subject (36 years 6 months old) was enrolled in the 5-day artemether-lumefantrine arm (*P. falciparum* 320/µL; Hb 8.8; EGA at enrolment 34 Weeks); a placental abruption occurred at 40 weeks, six weeks and two days after drug administration. The mother experienced pain and severe bleeding at home and arrived at hospital 24 hours later with no audible foetal heart-beat. The event was classified as not related to the study drug.
4) The subject (33 year 3 months old) was enrolled in the 2$^{nd}$ trimester and treated with 3-days artemether-lumefantrine L (*P. falciparum* 334/µL; Hb 10.4; EGA at enrolment 23 weeks). The SAE occurred 16 weeks after enrolment. The patient had a prolonged labor complicated by peripartum asphyxia and a genital infection. The baby had a low apgar

score at birth and his conditions worsened rapidly with fever and respiratory distress. The baby was monitored for ten days until complete recovery. The event was classified as not related to the study drug.

**Pharmacokinetic analysis methods**

Population-based nonlinear mixed-effects modelling was performed in the software NONMEM v7.3 (Icon Development Solutions, Ellicott City, Maryland, USA). Pirana v2.9.2 and Perl-speaks-NONMEM v4.6.0 were used to facilitate the modelling process (1). Graphical diagnostics were performed using R v3.2.0 (R Foundation for Statistical Computing), RStudio v0.98.1103 and Xpose v4 (2). Model discrimination was based on the objective function value (OFV), proportional to −2×log-likelihood of the data, and a drop of at least 3.84 or 6.64 was considered significant at 0.05 and 0.01, respectively, when adding one parameter in a nested model. All concentration measurements were converted into their natural logarithms. Artemether and DHA concentration-time data were fitted simultaneously, while lumefantrine and desbutyl-lumefantrine were modelled sequentially. Complete (100%) in vivo conversion of parent drug to metabolite was assumed for both artemether and lumefantrine. In the sequential approach, the structural pharmacokinetic lumefantrine model and individual parameter estimates were imputed into the dataset when fitting DBL concentration-time data. One-, two- and three-compartment disposition models were evaluated for all compounds. First-order absorption and a transit compartment absorption model with 1–10 fixed transit compartments were evaluated to describe the absorption of artemether and lumefantrine (3). Relative bioavailability was fixed to unity but implemented to allow for between-subject variability in the absorption of administered drugs. Between-subject variability was evaluated on all pharmacokinetic parameters, using an exponential function.

Between-occasion variability (i.e. within-subject variability between dose occasions) were evaluated on all absorption parameters for artemether and lumefantrine (i.e. relative bioavailability and mean transit time), and on artemether elimination clearance to assess the presences of enzyme auto-induction. An additive residual error on log-transformed data (essentially equivalent to an exponential error on normal scale data) was used to describe the unknown variability. Data below the LLOQ were handled either with the M1 method (omitting the data) or the M3 method (treating the data below LLOQ as categorical data). The impact of body weight on the pharmacokinetic parameters was evaluated by adding it as an allometric function to all clearance (power of 0.75) and volume (power of 1) parameters. Other covariates (haemoglobin, haematocrit, treatment duration, ALT and AST levels at baseline, and pregnancy) were evaluated in the lumefantrine and artemether-DHA models. All continuous covariates were implemented as linear effects and evaluated in a stepwise manner using a forward addition ($p<0.05$) and backward elimination ($p<0.001$) approach. Categorical covariates were evaluated as proportional effects. Potential pregnancy effects were evaluated both as categorical (non-pregnant vs pregnant), categorical with three categories (non-pregnant, 2$^{nd}$ trimester, 3$^{rd}$ trimester), and as continuous (EGA) covariates. Pregnancy effects on primary and secondary pharmacokinetic parameters were also assessed for the active compounds (DHA and lumefantrine) using a full covariate approach. Pregnancy was added simultaneously as a categorical covariate on all parameters except relative bioavailability (due to identifiability issues), and the pregnancy effect on secondary parameters (terminal elimination half-life, maximum concentration, area-under the curve, and time to maximum concentration) were evaluated using a bootstrap approach (200 resampled datasets). Dose regimen (3-day vs. 5-day treatment) was evaluated as a categorical

covariate on all pharmacokinetic parameters. Other covariates investigated were enrolment parasitaemia, haemoglobin and haematocrit.

Model diagnostics were performed using goodness-of-fit plots and visual predictive checks (2000 simulations). Relative standard error (precision) of model parameters were calculated using the Sampling Importance Resampling function in Perl-Speaks-NONMEM (4). NONMEM was used to compute η and ε shrinkages (5) and provide secondary parameters (terminal elimination half-life, maximum concentration, area-under the curve, and time to maximum concentration and, for lumefantrine, day 7 concentrations and time above 280 ng/mL). The relationship between observed QTc and model predicted lumefantrine concentrations was evaluated with ordinary linear regression (GraphPad Prism v8). Ordinary linear regression was also used to investigate the relationship between model predicted drug exposure and parasite clearance half-life.

**References**


1. Lindbom L, Ribbing J, Jonsson EN. 2004. Perl-speaks-NONMEM (PsN)--a Perl module for NONMEM related programming. Comput Methods Programs Biomed 75:85-94.
2. Jonsson EN, Karlsson MO. 1999. Xpose--an S-PLUS based population pharmacokinetic/pharmacodynamic model building aid for NONMEM. Comput Methods Programs Biomed 58:51-64.
3. Savic RM, Jonker DM, Kerbusch T, Karlsson MO. 2007. Implementation of a transit compartment model for describing drug absorption in pharmacokinetic studies. J Pharmacokinet Pharmacodyn 34:711-26.
4. Dosne AG, Bergstrand M, Karlsson MO. 2017. An automated sampling importance resampling procedure for estimating parameter uncertainty. J Pharmacokinet Pharmacodyn 44:509-520.
5. Savic RM, Karlsson MO. 2009. Importance of shrinkage in empirical bayes estimates for diagnostics: problems and solutions. AAPS J 11:558-69.

## Supplementary tables

**Table S1.** Median dose of artemether (AM) and lumefantrine (LUM) administered by treatment arm

| Treatment | Group | N | Median dose AM mg/kg (min-max) | Median dose LUM mg/kg (min-max) |
|---|---|---|---|---|
| **Manufacturer target dose** | | | *5-24 mg/kg* | *29-144 mg/ kg* |
| **3-day** | 2nd trimester | 12 | 8.35 (5.85-10.67) | 50.09 (35.12-64.00) |
| | 3rd trimester | 12 | 7.17 (5.65-10.91) | 42.99 (33.88-65.45) |
| | Non-pregnant | 24 | 9.23 (7.27-11.56) | 55.38 (43.64-69.33) |
| | Total | 48 | 8.57 (5.65-11.56) | 51.43 (33.88-69.33) |
| **5-day** | 2nd trimester | 12 | 14.55 (8.89-15.38) | 87.27 (53.33-92.31) |
| | 3rd trimester | 12 | 11.85 (10.39-16.00) | 71.12 (62.34-96.00) |
| | Non-pregnant | 24 | 14.68 (8.42-18.18) | 88.08 (50.53-109.09) |
| | Total | 48 | 13.45 (8.42-18.18) | 80.68 (50.53-109.09) |

**Table S2.** Absolute and relative mean haematocrit (SD) difference day 0 to 7 and day 0 to 28, by treatment arm

| Parameters | Timepoint | Non-pregnant women | | p-value |
|---|---|---|---|---|
| | | **3-day** | **5-day** | |
| **Absolute Hct difference** | Day 0 - 7 | N=24; 0.38 (2.41) | N=24; 0 (2.32) | 0.34 |
| | Day 0-28 | N=24; -0.13 (1.94) | N=23; 0.04 (2.14) | 0.95 |
| **Relative Hct difference** | Day 0 - 7 | N=24; -0.70 (6.81) | N=24; 0.24 (6.27) | 0.32 |
| | Day 0 -28 | N=24; 0.70 (5.71) | N=23; 0.03 (6.06) | 0.96 |
| **Parameters** | **Timepoint** | **Pregnant women** | | **p-value** |
| | | **3-day** | **5-day** | |
| **Absolute Hct difference** | Day 0 - 7 | N=24; -1.3 (2.4) | N=24; 0.5 (2.8) | 0.07 |
| | Day 0-28 | N=24; -1.9 (2.7) | N=24; -0.8 (3.5) | 0.21 |
| **Relative Hct difference** | Day 0 - 7 | N=24; 4.5 (8.4) | N=24; -1.3 (8.6) | 0.08 |
| | Day 0 -28 | N=24; 6.9 (9.7) | N=24; 3.2 (11.6) | 0.16 |

## Supplementary Figures

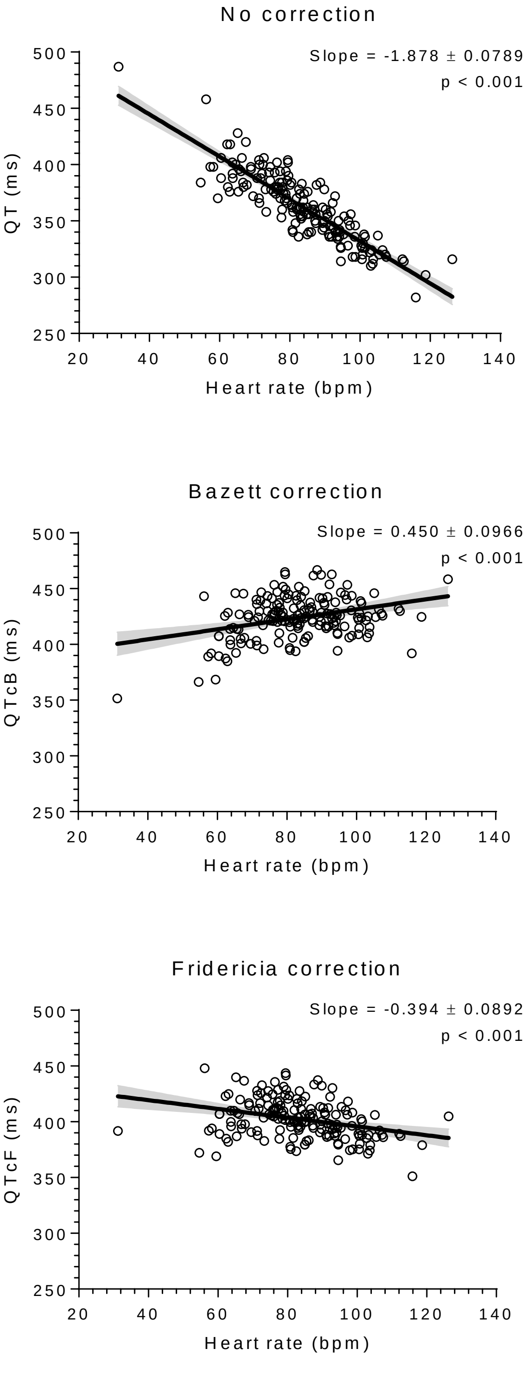


**Figure S1.** Evaluation of correction factors. Correlation between uncorrected QT interval and heart rate (upper), Bazett-corrected QT interval and heart rate (middle), and Fridericia-corrected QT interval and heart rate (lower).The solid line is the mean regression of observed data and the shaded area show the 95% confidence interval of this estimate. The slope is given as the estimated value ± standard error. The p-value indiciate the probability that this slope is different from zero-slope

Lumefantrine/Desbutyl-lumefantrine

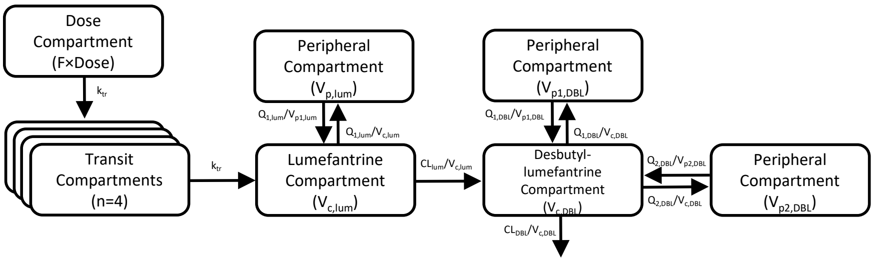


Artemether/Dihydroartemisinin

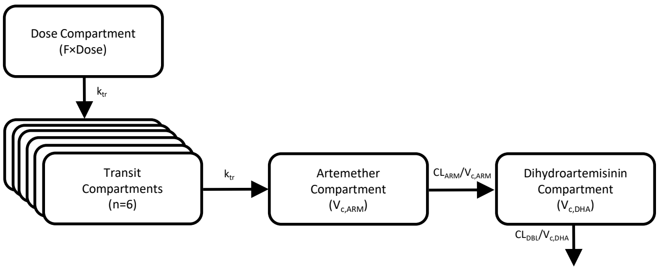


**Figure S2.** Graphical representation of the pharmacokinetic models. F is the relative bioavailability. $k_{tr}$ is the absorption rate constant for the absorption through the transit compartments. $CL_{LUM}$, $CL_{DBL}$, $CL_{AM}$, and $CL_{DHA}$ are the elimination clearances of lumefantrine, desbutyl-lumefantrine, artemether and DHA respectively. $V_{C,LUM}$, $V_{C,DBL}$, $V_{C,AM}$, AND $V_{C,DHA}$ are the central volume of distributions of lumefantrine, desbutyl-lumefantrine, artemether and DHA, respectively. $Q_{1,LUM}$ is the inter-compartment clearance to LUM peripheral compartment. $V_{P,LUM}$ is the volume of distribution. $Q_{1,DBL}$ and $Q_{2,DBL}$ are the inter-compartmental clearances for desbutyl-lumefantrine first and second peripheral compartments, respectively. $V_{p1,DBL}$ and $V_{p2,DBL}$ are the volume of distribution for DBL first and second peripheral compartments, respectively

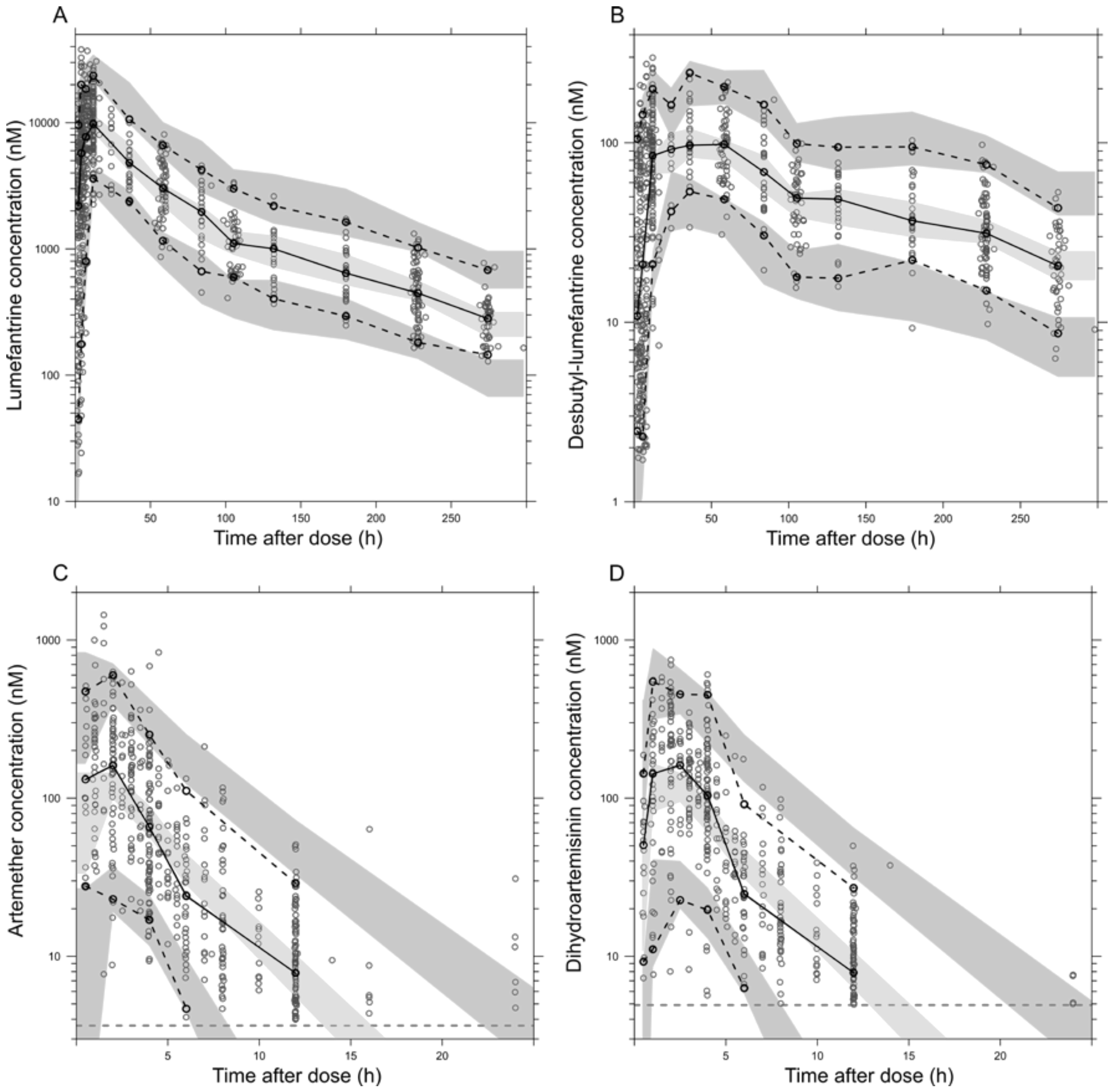


**Figure S3.** Visual predictive check of the final population pharmacokinetic model for lumefantrine (A), desbutyl-lumefantrine (B), artemether (C), and dihydroartemisinin (D). Open circles represent observed concentrations and solid lines represent the 5th, 50th, and 95th percentiles of the observed data. The shaded areas represent the 95% confidence intervals around the simulated 5th, 50th, and 95th percentiles

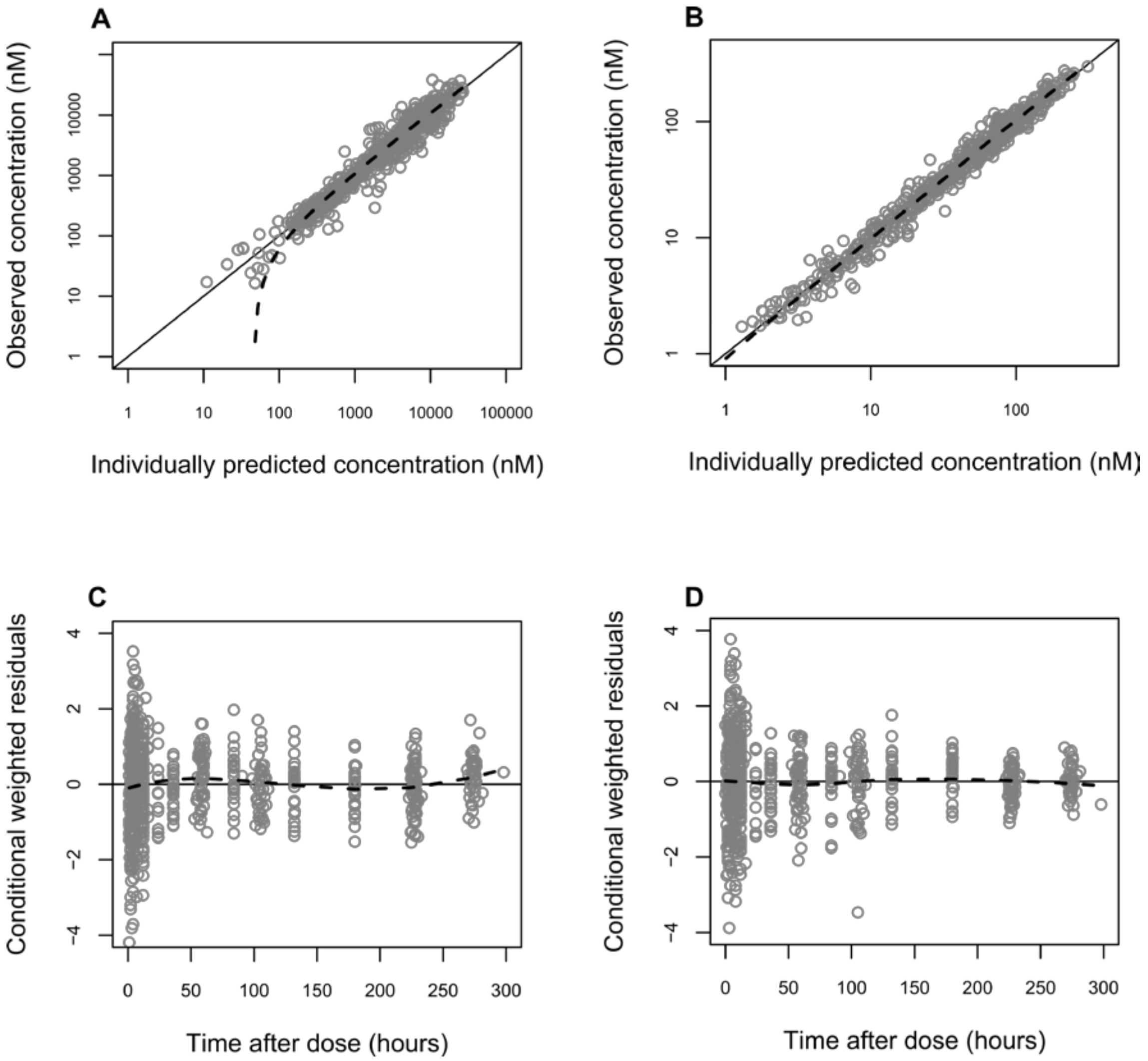


**Figure S4.** Basic goodness-of-fit plots of the final model for lumefantrine (A,C) and desbutyl-lumefantrine (B,D). Observed concentrations plotted against individually predicted concentrations of lumefantrine (A) and desbutyl-lumefantrine (B). Conditional weighted residuals plotted against time after dose for lumefantrine (C) and desbutyl-lumefantrine (D). The solid line is the line of identity. The dashed line represents the locally weighted least square regression line.

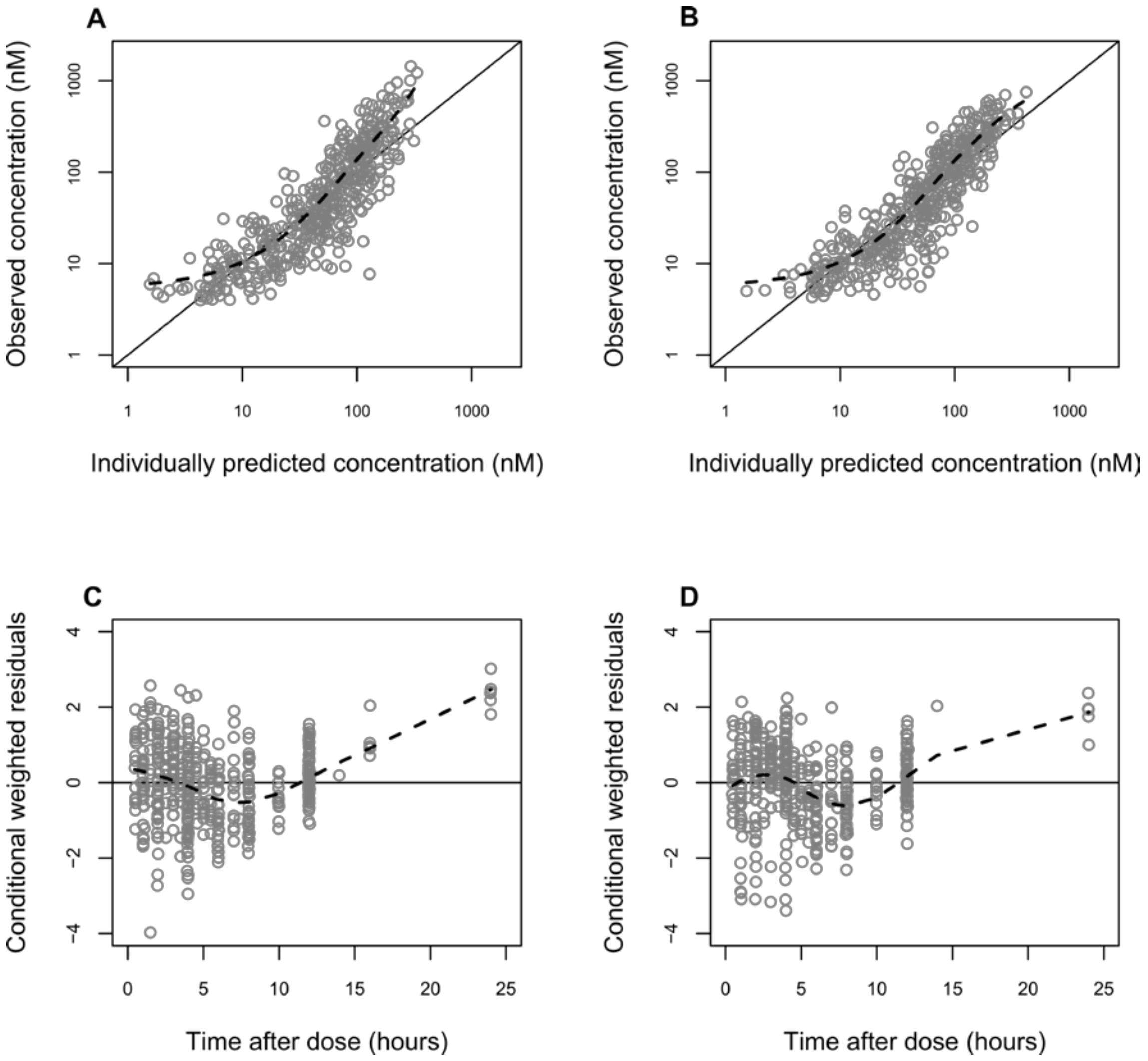


**Figure S5.** Basic goodness-of-fit plots of the final model for artemether (A,C) and DHA (B,D). Observed concentrations plotted against individually predicted concentrations of artemether (A) and DHA (B). Conditional weighted residuals plotted against time after dose for artemether (C) and DHA (D). The solid line is the line of identity. The dashed line represents the locally weighted least square regression line.
